# A Retrospective Analysis of Applications for Registration of Generic Medicines Processed by the Medicines Control Authority of Zimbabwe

**DOI:** 10.1007/s43441-022-00469-y

**Published:** 2022-10-21

**Authors:** Brilliant Tinashe Samunda, Tariro Sithole, Star Khoza

**Affiliations:** 1 Medicines Control Authority of Zimbabwe, 106 Baines Avenue, Avenues, Harare, Zimbabwe; 2 University of the Western Cape, Robert Sobukwe Rd, Bellville, Cape Town, 7535 South Africa

**Keywords:** Generic medicines, Deficiencies, Retrospective analysis, MCAZ, Review timelines

## Abstract

**Background:**

Many applications for registration of medicines are rejected because applicants fail to submit or resolve critical deficiencies in the quality, efficacy, and safety of the medicines. The study aimed to establish approval rates, processing timelines, and common deficiencies of generic medicines applications processed by the Medicines Authority of Zimbabwe (MCAZ).

**Method:**

A retrospective study of applications finalized by MCAZ between 2018 and 2020 was conducted. Data were collected from the assessment reports and verified with copies of letters sent to the applicants. Deficiencies were classified as administrative, quality, efficacy, and safety. Other characteristics collated included time to finalization, dosage form, region of origin, and therapeutic class.

**Results:**

Of the 579 finalized applications, 74.1% were approved while 25.9% were refused. Approved applications had more review cycles (median = 3 cycles) compared to refused applications (median = 2 cycles). However, refused applications had longer review times (median = 25 months) compared to approved applications (median = 18 months). The majority of applications (83.0%) were from Asian manufacturers and intended for oral administration (66.1%). Medicines for the endocrine system (50.0%) and rheumatism/gout (53.3%) had lower approval rates compared to other therapeutical classes (*p* < 0.001). The most common reasons for refusal of applications included failure to respond to review queries (52.6%), deficiencies in the API information (54.7%), FPP specifications (42.7%), FPP stability data (36.0%), and pharmaceutical development (31.3%).

**Conclusion:**

To improve the quality of applications and evaluation outcomes, there may be a need for the regulatory authority to engage applicants through training and pre-submission meetings.

## Introduction

Generic medicines are crucial to public health in many ways [1]. The cost advantage over innovator products does not only lead to improved access to medicines but also promotes patient compliance which in turn enhances treatment outcomes [2, 3]. The effective and transparent regulation of generic medicines increases the trust of patients, and healthcare professionals [3]. National Medicines Regulatory Authorities (NMRA), such as the Medicines Control Authority of Zimbabwe (MCAZ), play a critical role in ensuring that generic medicines on the market are effective, safe, and of good quality.

Applications for registration include a detailed dossier describing the medicine’s quality, safety, and efficacy in Common Technical Document (CTD) format consisting of Modules 1 to 5. After submission, the application goes through the screening process and a response is sent within 90 days of receipt. In cases where the product fails the screening, the applicant is required to submit additional information before a substantive review of the application [4]. Product applications that pass screening progress to the evaluation stage. Queries and deficiencies noted during the review process are communicated to the applicants with the expectation that all critical deficiencies will be resolved within two months [5]. The final assessment decision which can either be an approval or refusal is usually made after at least one review cycle. Rejection of an application is usually an indication that information submitted to the NMRA is considerably incomplete or unacceptable [1].

Despite the availability of guidelines for the submission of applications for registrations, many applicants find it difficult to prepare and submit acceptable applications [1]. The high proportion of rejected applications is attributed to the failure of applicants to resolve critical deficiencies highlighted during the review process [5]. Submitting unsatisfactory or incomplete applications does not allow a substantive review and the process of “repairing” the application results in an extended review period. This is usually inefficient and wasteful of resources for both the applicant and the NMRA [6]. In some cases, the applicant may have to review the entire manufacturing process or re-develop a product [2]. Although the deficiencies in some of the failed applications may be successfully addressed in resubmissions, delayed approvals may limit the treatment choices [2, 7].

Some regulatory agencies such as European Medicines Agency (EMA) and Therapeutic Goods Administration (TGA) in Australia, as part of their commitment to transparency, publish information relating to assessments of applications via public assessments reports for approved applications [8]. Despite transparency being highlighted as an important aspect in the effective regulation of medicines, comprehensive information on finalized applications is often not available in the public domain [8]. This prevents the analysis of the most important factors associated with approval or refusal of applications for registrations [2]. Availability of information on factors associated with assessment outcomes can lead to a better understanding of the regulator’s expectations by the applicants [9]. The objective of the study was to analyze applications for registrations of generic medicines finalized by the MCAZ from 2018 to 2020 to establish approval rates, processing timelines, and common deficiencies.

## Materials and Methods

This was a retrospective study to analyze market authorization applications for generic medicines finalized by the MCAZ between January 1, 2018, and December 31, 2020. Finalized applications were defined as applications that were either refused or approved by MCAZ during the study period. Biosimilars, complementary medicines, and veterinary medicines were excluded from the study. Registration committee meeting minutes were used to verify the final decisions for each application. The data was anonymized by removing the potential identifiers.

Deficiencies highlighted in the assessment reports were verified with those communicated to the applicants/manufacturers using copies of the letters sent to the applicants. Deficiencies were classified as administrative, quality, efficacy, and safety. Quality deficiencies were defined as inadequate/unsatisfactory information in the chemistry, manufacturing, and controls (CMC) for both the Active Pharmaceutical Ingredient (API) and Finished Pharmaceutical Product (FPP). Efficacy deficiencies were defined as failure to demonstrate the efficacy of the drug for the proposed treatment indication. Safety deficiencies were defined as failure to demonstrate that the medicine had no adverse drug events that can lead to a negative benefit-risk profile. The data was compiled on Microsoft Office Excel sheet and analyzed using IBM SPSS Statistics 23. Bivariate analysis was conducted using the student’s t-test and the Mann–Whitney test for continuous variables and Chi-square analysis for categorical variables, as appropriate. A significance level of alpha = 0.05 was set a priori for all statistical tests. The study did not include human subjects, therefore an exemption of ethical review (MRCZ/3/303) was granted by the Medical Research Council of Zimbabwe (MRCZ).

## Results

A total of 579 applications finalized by the MCAZ during the period 1st January 2018 to 31st December 2020 were analyzed. Out of these, 429 (74.1%) applications were approved while 150 (25.9%) were refused. These products went through at least one review cycle except for one application which was refused after screening before a substantive review. It was noted that approved applications (median = 3 cycles) had more review cycles compared to refused applications (median = 2 cycles). Nonetheless, it took more time to finalize the refused applications (Median = 25 months) than approved applications (median = 20 months) as shown in Table 1.

**Table 1:** Comparison of processing timelines for approved and refused applications

	All applications	Approved (*N* = 429)	Rejected application (*N* = 150)	*p* value
Processing time, months (Median)	20 (13–28)	18 (11–26)	25 (18–36)	< 0.001

Even though most of the finalized applications were meant for oral administration (66.1%), topical formulations had the highest approval rate (93.1%) compared to the other dosage forms. Upon classification in terms of therapeutic class, most finalized applications (24.7%) were for anti-infective medicines. However, applications for analgesics had the highest approval rate (84.2%). These analyzed applications were manufactured in 25 different countries located in Africa (7.8%), Asia (83.0%), and Europe (9.2%%). Further grouping showed that 53 (9.2%) manufacturers were from the International Council for Harmonisation of Technical Requirements for Pharmaceuticals for Human Use (ICH) countries while 526 (90.8%) of the applications originated from non-ICH countries. The comparison between refused and approved applications is shown in Table 2.

**Table 2:** Comparison of approval rates by dosage form, therapeutic class, and region of origin

Dosage forms	All applications (*N* = 579)	Approved (*N* = 429) *n* (%)	Refused (*N* = 150) *n* (%)	*p* value
Inhalations	13	10 (76.9)	3 (23.1)	0.094
Injectables	154	109 (70.8)	45 (29.2)	
Oral formulations	383	283(73.9)	100 (26.1)	
Topical formulations	29	27 (93.1)	2 (6.9)	

Therapeutic class	All applications	Approved *n* (%)	Refused *n* (%)	*p* value
Analgesics	38	32 (84.2)	6 (15.8)	< 0.001
Anti-infective medicines	143	115 (80.4)	28 (19.6)	
Cardiovascular and blood medicines	97	69 (71.1)	28 (28.9)	
Central nervous system medicines	56	33 (58.9)	23 (41.1)	
Endocrine system medicines	18	9 (50.0)	9 (50.0)	
Gastrointestinal medicines	35	21 (60.0)	14 (40.0)	
Medicines acting on the respiratory tract	30	20 (66.7)	10 (33.3)	
Medicines used in rheumatism and gout	30	16 (53.3)	14 (46.7)	
Other	132	114 (86.3)	18 (13.7)	

Region of origin	All applications	Approved *n* (%)	Refused (*n* = 150)	
Africa (Non-ICH)	45	35 (77.8)	10 (22.2)	< 0.001
Asia (Non-ICH)	481	343 (71.3)	138 (28.7)	
Europe (ICH)	53	51 (96.2)	2 (3.8)	

There was no relationship between the regulatory decision and the intended route of administration (*p* value = 0.094). On the other hand, it was noted that the therapeutic class and region of origin of the FPP manufacturing site had a significant association with the regulatory decision (*p* value < 0.001). Applications from non-ICH regions (Asia 28.7% and Africa 22.2%) had higher refusal rates compared to those from the ICH region (Europe 3.8%). Analgesics (84.2%) and anti-infectives (80.4%) had higher approval rates compared to medicines for rheumatism and gout (53.3%) and endocrine system medicines (50.0%).

### Common Deficiencies in Refused Applications

A total of 22 deficiencies were observed in refused applications. The deficiencies were cutting across the 5 modules of the dossier with the majority emanating from the quality section (module 3). The top five deficiencies were mainly due to either inadequate or unacceptable API information (Sect. 3.2.S.1 to 3.2.S.6) (53.0%), FPP specifications (42.7%), FPP stability data (36.0%), Pharmaceutical development (31.3%) and FPP manufacturer cGMP non-compliance (28.7%). All the deficiencies were noted to have a significant association with the regulatory decision (refusal) except for API forced degradation and non-clinical data (*p* > 0.05). Further analysis showed a significant relationship between failure to submit additional data within the stipulated timelines and refusal of the applications (*p* < 0.001) (Table 3).

**Table 3:** Distribution of critical deficiencies among refused products

Section of dossier	Critical deficiencies	*n* (%)	*p* value
Administrative			
Module 1.2	Complete MC8 form	14 (9.3%)	< 0.001
Module 1.2.5.2	Certificate of Pharmaceutical Product	13 (8.7%)	< 0.001
Module 1.6	API manufacturing site GMP compliance	36 (24.0%)	< 0.001
Module 1.6	FPP manufacturing Site GMP compliance	43 (28.7%)	< 0.001
Failure to respond to request for additional data within the stipulated timelines		79 (52.6%)	< 0.001
Quality			
Module 3.2.S.1–6	API information (CEP/DMF)	82 (54.7%)	< 0.001
Module 3.2.S.7.1	API Forced degradation Studies	2 (1.3%)	0.067
Module 3.2.S.7.3	API Stability Data	38 (25.3%)	< 0.001
Module 3.2.P.1	Dosage Unit Batch Formulae	6 (4.0%)	< 0.001
Module 3.2.P.2	Pharmaceutical development	47 (31.3%)	< 0.001
Module 3.2.P.4.1	Excipient Specification	7 (4.7%)	< 0.001
Module 3.2.P.5.1	FPP Specifications	64 (42.7%)	< 0.001
Module 3.2.P.5.2	Analytical Procedures	18 (12.0%)	< 0.001
Module 3.2.P.5.3	Analytical method validation	16 (10.7)	< 0.001
Module 3.2.P.3.3	FPP manufacturing Procedure	35 (23.3%)	< 0.001
Module 3.2.P.3.5	Manufacturing Process Validation	30 (20.0%)	< 0.001
Module 3.2.R.1	BMR	31 (20.7%)	< 0.001
Module 3.2.P.8.3	FPP Accelerated Stability Data	30 (20.0)	< 0.001
Module 3.2.P.8.3	FPP Long term stability Data	54 (36.0%)	< 0.001
Safety and efficacy			
Module 5	Bioequivalence study	36 (24.0%)	< 0.001
Module 5	Biowaiver applications	19 (12.7%)	< 0.001
Module 5	Summary of clinical studies	8 (5.3%)	< 0.001
Module 4	Non-Clinical data	1	0.259

## Discussion

The approval rate for most dosage forms was generally above the overall approval rate (74.1%) except for injectables (70.8%) and oral formulations (73.9%). This could be due to the additional requirements that are specific to these dosage forms. Sterility is critical for the injectable formulation, and it is achieved by controlling several factors such as the sterilization procedure, the integrity of the container closure system, and bioburden [10]. On the other hand, applications for oral formulation especially poorly soluble molecules require bioequivalence studies or biowaiver applications. These requirements are currently not applicable to most topical and inhalation formulations.

The region of origin was an important independent predictor of the outcome of applications for registration, with the highest approvals observed in applications from ICH regions. Applications originating from ICH are usually established products that have already been subjected to rigorous review processes in their countries of origin, hence the major deficiencies would have been addressed before they are submitted to MCAZ. On the other hand, applications originating from non-ICH regions with fewer international registrations usually will have more critical issues that might need to be addressed before approval.

Although approved applications (median 3 cycles) had more review cycles compared to refused applications (median 2 cycles), refused applications were open for a longer duration (median: 18 months vs 25 months). The delay in the finalization of refused products could not be attributed to either the applicant or the regulator because there was no tracking mechanism by the time of the cut-off date of analysis (31 December 2020). In order to track regulators’ and applicants’ times, the MCAZ recently implemented the stop clock and start closure mechanism [4]. This is in line with other regulatory agencies such as Health Canada and European Medicines Agency (EMA) that have also implemented the same tracking mechanism to allow for the pausing of the application review clock to accurately reflect the time spent by the regulator on the product vs the time spent by the applicant to respond to queries for additional data request [11].

It was noted that most applications (52%) were refused because of failure to respond to additional data requests within the stipulated deadlines. One of the possible reasons for this is that applicants were not able to address the issues raised by the MCAZ hence they opted not to respond. In Europe, applicants faced with the probability of refusal of the applications for registration often prefer to withdraw their applications. This is reflected in the analysis done for the period between 2004 and 2007, where more than 90% of applications that received a negative outcome from the EMA were due to withdrawals [12]. Another reason could be the minimum interactions between the regulator and applicants during the evaluation process. Lack of close communication between applicants and the EMA has also been pointed out as a significant contributing factor to the failure of Applications [2]. Currently, it is not mandatory for the applicant to request scientific advice from MCAZ. Nonetheless, previous studies have shown that formal meetings between the regulator and sponsors during the review process have an impact on approval rates and are associated with shorter review times [12].

### Common Deficiencies

#### Current Good Manufacturing Practices (cGMP)

While it is the responsibility of the manufacturer to ensure cGMP compliance at the manufacturing site, applicants have a clear role in facilitating GMP compliance before the submission of their applications [13]. Despite the availability of MCAZ published guidelines for Good Manufacturing Practice, some applicants still find it hard to meet the cGMP requirements. In this study, it was observed that 28.7% and 24% of the refused applications had outstanding GMP issues relating to the FPP and API manufacturing sites respectively. The majority of these applications had expired GMP certificates while some lacked the proof of cGMP. Currently, MCAZ does not inspect API manufacturing sites, therefore, submission of proof of cGMP compliance issued by regulatory authorities from ICH countries or National Regulatory Authorities (NRAs) is sufficient. However, the approach used for FPP manufacturing sites is somewhat different. Applications are only approved once the FPP manufacturer has been deemed to be cGMP compliant by the MCAZ. Exemptions from MCAZ GMP inspections are only awarded to manufacturing sites under the jurisdiction of SRAs in ICH countries. In some cases, reliance through desk review may be used to clear manufacturing sites inspected by SRAs and World Health Organization (WHO) [14].

#### Active Pharmaceutical Ingredient

Issues raised under the API sections were mostly related to either inadequate or unacceptable details on the manufacturing process (3.2.S.2), control of drug substances (3.2.S.4), and stability (3.2.S.7). These deficiencies were comparable to the common API deficiencies observed by other agencies. Table 4 shows a comparison of common deficiencies among different agencies. The observation is expected since more information is required in these sections of the CTD compared to other sections such as general properties (3.2.S.1), reference standards (3.2.S.5), and container/closure system (3.2.S.6).

**Table 4:** Comparison of the most common API and FPP deficiencies from other agencies [27, 28]

Active pharmaceutical ingredient (API)				
MCAZ	SAHPRA	TFMDA	EDQM	WHOPQTm
3.2. S.4.1	3.2. S.3.1	3.2. S.2.2	3.2. S.2.3	3.2.S.2.3
3.2. S.2.2	3.2.S.1 & 3	3.2. S.2.3	3.2. S.3.2	3.2.S.3.2
3.2. S.7	3.2.S.4.1 & 3	3.2. S.4.1	3.2. S.2.2	3.2.S.2.3
3.2. S.5	3.2.S.7.1 &3	3.2. S.4.3	3.2. S.2.4	3.2.S.2.4
3.2.S.1&3	3.2. S.2.2	3.2. S.7	3.2. S.4.4	3.2.S.4.1 & 5

Finished pharmaceutical product (FPP)				
MCAZ	SAHPRA	TFMDA	EMA	WHOPQTm
3.2. P.5.1	3.2.P.5.1	3.2. P.5.1	3.2. P.5	3.2. P.3
3.2. P.8.3	3.2.P. 3.3	3.2. P.5.3	3.2. P.3	3.2. P.4
3.2. P.2	3.2. P.1	3.2. P.3.3	3.2. P.2	3.2. P.5
3.2. P.3.3	3.2.P.8/3	3.2. P.3.5	3.2. P.8	3.2. P.8
3.2. P.3.5	3.2. P.7	3.2. P.6	3.2. P.4	3.2. P.7

Modules: 3.2.S.1 general properties of the API, 3.2.S.2 manufacture, 3.2.S.2.2 description of manufacturing process and process controls, 3.2.S.2.3 control of materials, 3.2.S.2.4 controls of critical steps and intermediates, 3.2.S.3 characterization, 3.2.S.3.2 impurities, 3.2.S.4 control of the API, 3.2.S.4.1 specifications, 3.2.S.4.4 batch analysis, 3.2.S.5 reference materials, 3.2.S.7 Container closure system, 3.2.S.7 stability, Modules: 3.2.P.1 Composition and Description, 3.2.P.2 Pharmaceutical Development, 3.2.P.2.2 Pharmaceutical Development, 3.2.P.3.3 Description of the Manufacturing Process, 3.2.P.3.5 Process Validation or Evaluation, 3.2.P.4 Control of the IPIs, 3.2.P.5 Control of FPP, 3.2.P.5.1 Specifications, 3.2.P.7 Container Closure System, 3.2.P.8 Stability Data

Data covering the manufacturing process, control of materials, and proposed starting materials included in most applications was neither adequate nor satisfactory to meet the CTD requirements. Such information on drug substance manufacturing is critical and affects subsequent sections of the dossier such as 3.2.S.3.2 Impurities and 3.2.4.1 API specifications. This information is usually submitted separately as part of the restricted part of the Drug Master File (DMF) by the API manufacturer upon request.

Regarding the control of the drug substance, the typical issues included the exclusion controls for impurities such as related substances and residual solvents without proper scientific justification. It is recommended that specifications for drug substances listed in pharmacopeia should confirm by the pharmacopeia [15]. Any other impurity listed in other international pharmacopeias should also be controlled in the API specifications unless otherwise, scientific justification should be provided. Limits for impurities for non-compendial APIs should be in line with the relevant ICH guidelines [16–19].

To avoid some of the API deficiencies highlighted in this study, applicants may use APIs with a certificate of suitability (CEP) or confirmation of WHO API prequalification (CPQ) when necessary. Applications supported by current CEPs or CPQs are exempted from including information from certain sections of module 3.2.S, as highlighted in the guideline on submission of documentation for registration of a multisource (generic) Finished Pharmaceutical Product (FPP): quality part in the Common Technical Document (CTD) format [20]. In a study conducted in Taiwan (2016), it was noted that applications with full API information had high chance of being rejected and were characterized by longer review times compared to those supported by CEPs with abbreviated API sections [21].

Some agencies such as the ANVISA (Agência Nacional de Vigilância Sanitária) now require pre-market approval of APIs before an FPP is approved [22]. MCAZ may need to consider the registration and publishing of approved API sources after consultations with relevant stakeholders. This may eliminate the submission of applications with poor-quality APIs. API vendor selection is critical and has a significant impact on the drug product quality and its ability to comply with global regulatory requirements [22, 23].

### Finished Pharmaceutical Product

Issues raised from the drug product specifications were somewhat similar to those observed under the drug substances. The most common deficiencies were emanating from 3.2.P.5.1 FPP specifications, 3.2.P.8.3 FPP stability, 3.2.P.2 Pharmaceutical development, 3.2.P.3.3 Description of the manufacturing process and process controls, and 3.2.P.3.5 Process validation. The same issues were also pointed out as most prevalent in similar studies as summarized in Table 4. Critical issues observed for FPP specifications included inappropriate acceptance criteria e.g., wide limits for assay and omission of some critical tests e.g., related substances [15].

Zimbabwe falls under ICH Climatic Zone IVb, therefore stability data must demonstrate drug product stability throughout the proposed shelf life under the same conditions [20]. At the time of submission, the applicant is expected to submit 6 months of accelerated stability data and at least 12 months long-term stability [2, 20]. Notable issues under the drug product stability section included submission of stability data conducted under unacceptable ICH climatic conditions, container closures, and omission of some of the stability-indicating parameters such as appearance, assay, and related substances as highlighted by WHO [24]. Out of specifications and significant changes in the drug product were observed in a few applications. Some of the applications were lacking in-use stability data and stability data conducted at inverted positions, particularly for injectables.

To avoid such deficiencies, FPP manufacturers are recommended to follow the MCAZ guidelines, pharmacopeias, and ICH guidelines criteria where applicable. In cases where there is a deviation from the norm, scientific justification should always be included in the applications.

### Bioequivalence (BE) Studies

Under the BE section, the most recurring issues were related to the bioanalytical method and method validation. Similar to the study conducted to investigate common deficiencies in BE submission in ANDA assessed by USFDA, common issues raised included the lack of SOPs, and inadequate or absence of long-term stability data for the analytical samples [25]. Another study highlighted the submission of mean concentration data instead of concentration for every subject at every time point as analyzed in the BE studies and the lack of complete chromatograms from 20% of subjects as the common issues related to the bioanalytical report included [26]. Less frequent issues noted included lack of information on reference products (proof of purchase, carton labels, and storage conditions during transportation), absence of statistical output, and inadequate washout periods.

To bridge the gap on issues related to bioequivalence studies, resources need to be channeled toward training. The MCAZ has been conducting training on bioequivalence studies targeted at both regulators and industries across the African continent. The training has resulted in an improvement in the quality of BE study submissions over the years.

## Conclusion

The high proportion of refused applications highlights a gap between the regulator and applicants/manufacturers’ expectations. The majority of the deficiencies that contributed to the refusal of these products are avoidable. There is a need for continuous engagement from both the regulator and the applicant to improve the quality of applications for registration submissions. Applicants must do their due diligence before submission of applications for registrations. If need be, resources and training should be channeled toward the critical areas highlighted in this study. On the other hand, technical assistance, and scientific advice from the MCAZ should be provided during the evaluation process especially to new applicants to avoid recurring deficiencies and the ultimate refusal of applications for registrations.

## Abbreviations

SAHPRA: South African Health Products Regulatory Authority
TFDA: Taiwan Food and Drug Administration
EDQM: European Directorate for the Quality of Medicines & HealthCare
EMA: European Medicines Agency
WHOPQTm: World Health Organisation Prequalification Team
